# Combinatorial Effects of Chrysin with Doxorubicin, 5-Fluorouracil, and Cyclophosphamide on Triple-Negative Breast Cancer Cell Line

**DOI:** 10.5812/ijpr-157446

**Published:** 2025-02-12

**Authors:** Sedighe Yosefi, Hamid Madanchi, Abbas Pakdel, Parviz Kokhaei, Maral Hemati, Negar Sarmadi, Majid Sirati-Sabet

**Affiliations:** 1 Department of Clinical Biochemistry, School of Medicine, Shahid Beheshti University of Medical Sciences, Tehran, Iran; 2 Department of Medical Biotechnology, Faculty of Medicine, Semnan University of Medical Sciences, Semnan, Iran; 3 Nervous System Stem Cells Research Center, Semnan University of Medical Sciences, Semnan, Iran; 4 Department of Immunology, School of Medicine, Arak University of Medical Sciences, Arak, Iran; 5 Cancer Research Center, Semnan University of Medical Sciences, Semnan, Iran; 6 Department of Biochemistry, Faculty of Medicine, Semnan University of Medical Sciences, Semnan, Iran

**Keywords:** Combinatorial Therapy, Chrysin, Cyclophosphamide, Doxorubicin, 5-Fluorouracil, Triple-Negative Breast Cancer

## Abstract

**Background:**

The primary challenges associated with chemotherapy treatment include the development of drug resistance. Chrysin (CH) has the potential to enhance the therapeutic efficacy of conventional chemotherapeutic agents. Additionally, CH, with its antioxidant properties, can reduce the side effects caused by reactive oxygen species (ROS) from chemotherapy.

**Objectives:**

This study focused on investigating the combination impact of CH with either 5-fluorouracil (5-FU), doxorubicin (DOX), or cyclophosphamide (CP) on the triple-negative breast cancer (TNBC) MDA-MB-231 cell line.

**Methods:**

Cytotoxicity was investigated using the MTT assay. The checkerboard microplate method was utilized to determine the effects of drug interactions. Apoptosis and cell cycle distribution were measured by flow cytometry. The classical scratch assay was used to examine cell migration ability.

**Results:**

The combination of 5-FU and DOX showed synergistic effects with CH (FIX < 1). Conversely, the interaction between CH and CP resulted in non-additive effects (FIX > 1). The combination treatment of CH with a chemotherapeutic drug was more effective in inducing early apoptosis than the drug alone and the control (P < 0.05). An increase in the sub-G1 phase was observed upon treatment with the combination of CH and chemotherapeutic drugs compared with the control and drugs alone (P < 0.05). Co-administration of CH with chemotherapeutic drugs induced a significant decrease in cell migration compared with the control and chemotherapeutic drugs alone (P < 0.05).

**Conclusions:**

The results revealed that combination therapy involving CH in conjunction with 5-FU and DOX demonstrated a more substantial therapeutic effect on TNBC cells than treatments with 5-FU and DOX individually.

## 1. Background

Triple-negative breast cancer (TNBC) represents a significant subgroup of breast cancer cases, accounting for up to 20% of all cases and having a considerable impact on cancer-associated mortality (1). The recurrence and mortality rates in TNBC patients remain higher than those of non-TNBC subtypes, with only about 30% of patients with TNBC achieving a complete response (2). Triple-negative breast cancer is characterized by impaired expression of progesterone and estrogen receptors and human epidermal growth factor receptor 2 (HER2), making it aggressive and unresponsive to targeted therapies (3). Therefore, conventional systemic chemotherapy remains the standard of care for nonsurgical TNBC.

During cancer treatment, the growth of resistant cancer stem cells (CSCs) contributes to chemotherapy resistance, leading to 90% of treatment failures (4). Chemoresistance is classified as primary, which is an inherent lack of response, or acquired, which develops during treatment through mechanisms such as increased drug efflux pumps, activation of DNA repair pathways, inhibition of cell death, and genetic or epigenetic changes (5). To overcome this, studies have explored a variety of strategies, such as targeted therapies and combination therapy with multiple chemotherapeutic agents and natural compounds (6). Moreover, chemotherapy is often accompanied by severe and undesirable side effects and related complications (7).

5-Fluorouracil (5-FU) is a synthetic pyrimidine used in cancer treatment as a thymidylate synthase inhibitor, but it can cause tissue toxicity (8). Doxorubicin (DOX) inhibits cancer cell growth by intercalating DNA and disrupting topoisomerase II, though its efficacy is limited by multidrug resistance (9). Cyclophosphamide (CP) is activated in the liver as a bifunctional alkylating agent that induces cell death by forming covalent bonds with DNA (10).

Research indicates that flavonoids like chrysin (CH), found in plants such as *Passiflora incarnata*, can reduce post-chemotherapy effects and enhance treatment efficacy due to their antiproliferative, anti-inflammatory, anti-tumor, and antioxidant properties (11). Chrysin inhibits tumor growth and induces apoptosis in breast cancer cell lines, with reported chemopreventive effects in various cancers and improved sensitivity to anticancer drugs (12). However, its specific mechanisms in breast cancer are not fully understood (13).

## 2. Objectives

In the present study, the therapeutic potential of CH in combination with 5-FU, DOX, and CP was evaluated on the MDA-MB-231 cell line, representing human TNBC cells.

## 3. Methods

### 3.1. Chemicals, Media, and Cell Line

Penicillin G/streptomycin, 3-(4,5-dimethylthiazol-2-yl)-2,5-diphenyltetrazolium bromide (MTT), dimethyl sulfoxide (DMSO), and CH were purchased from Sigma-Aldrich, USA. Annexin V/7-AAD was obtained from IQ Products, Netherlands. Cyclophosphamide, 5-FU, and DOX were sourced from GLS Pharma Ltd, India, and EBEWE Pharm, Austria, respectively. DMEM-F12 and fetal bovine serum (FBS) were purchased from Gibco. All other reagents were of the highest purity commercially available. The human TNBC cell line (MDA-MB-231, NCBI Code: C578) was obtained from the Pasteur Institute of Iran and cultured in DMEM-F12 culture medium supplemented with 10% FBS and 1% penicillin G/streptomycin, and incubated at 37°C in a 5% CO_2_ humidified incubator.

### 3.2. Cell Proliferation Assay

The cytotoxicity of the drugs was evaluated using the MTT assay with approximately 8 × 10^3^ cells cultured in 96-well plates. After 24 hours, cells were treated with varying doses of the drugs: Chrysin (13 - 800 µM), 5-FU (1.4 - 92 µM), DOX (0.09 - 6 µM), and CP (25 - 1600 µM). Cell viability was measured after 48 hours at 570 nm (14). The IC_25_ and IC_50_ values were calculated using non-linear fitting via GraphPad Prism software (GraphPad Software, San Diego, California, USA).

### 3.3. Checkerboard Method

The checkerboard microplate method was used to evaluate drug interactions in the MDA-MB-231 cell line. Drug dilutions were prepared according to each drug's IC_50_, with 8 × 10^3^ cells added per well and incubated for 48 hours at 37°C in a CO_2_ incubator. Cell growth was assessed using the MTT assay. Drug interactions were analyzed through fractional inhibitory concentrations (FIC), calculating FIC_A_ and FIC_B_ based on combined versus individual IC_50_ values. Fractional inhibitory concentrations A represents the IC_50A_ in combination divided by the IC_50A_ alone, and FIC_B_ denotes the IC_50B_ in combination divided by the IC_50B_ alone. After determining the IC_50 _of each drug, a linear dilution corresponding to the concentration range of 0.2 IC_50_ to 2 IC_50_ of each drug was used by making a 1:1 mixture of two drugs for calculating the FIC of each drug. A Fractional Inhibitory Index (FIX) was derived from FICA + FICB, where a FIX below 1 indicates a synergistic effect. A FIX value greater than 1 indicates a non-additive (antagonistic) effect.

### 3.4. Hemolytic Assay

A hemolysis assay was conducted to evaluate the hemocompatibility of CH with blood cells, using a 15% - 20% human erythrocyte suspension. Erythrocytes were incubated at 37°C with various CH concentrations (32 - 4000 μM) for 1 hour. Normal saline served as a negative control, while Triton X-100 was the positive control. Hemoglobin release was measured by absorbance at 415 nm using a microplate spectrophotometer (14).

### 3.5. Evaluation of Apoptosis

The apoptosis of MDA-MB-231 cells was evaluated via flow cytometry after 48 hours of treatment with 5-FU (6.2 µM), DOX (0.33 µM), and CP (192 µM), alone or with CH (94 µM). Cells were washed, resuspended in calcium-binding buffer, and stained with Annexin V-PE and 7-AAD. Apoptosis analysis was conducted using a Partec CyFlow space flow cytometer, with results processed using FlowJo software (15).

### 3.6. Cell Cycle Arrest Analysis

MDA-MB-231 cells were treated with 5-FU (6.2 µM), DOX (0.33 µM), and CP (192 µM) alone or in combination with CH (94 µM) for 48 hours. Subsequently, the cells were stained with a propidium iodide (PI) cocktail for 30 minutes at 4°C while kept in the dark. Cell cycle distribution analysis was then carried out using a BD FACS Calibur flow cytometer (USA) (15).

### 3.7. Cell Migration Assay

The migration of MDA-MB-231 cells was studied using a scratch assay after treatment with 5-FU (6.2 µM), DOX (0.33 µM), and CP (192 µM) alone or with CH (94 µM). Cell migration was observed at 24 and 48 hours, with gap sizes analyzed using Motic Images Plus software (16).

### 3.8. Statistical Analysis

The data were presented as mean ± SD. Values were compared using one-way and two-way ANOVA with GraphPad Prism (version 8.0.2, GraphPad Software, Inc., La Jolla, CA, USA). Differences were considered significant when P < 0.05. All tests were performed at least three times.

## 4. Results

### 4.1. Cytotoxicity Assay

**Figure 1.**
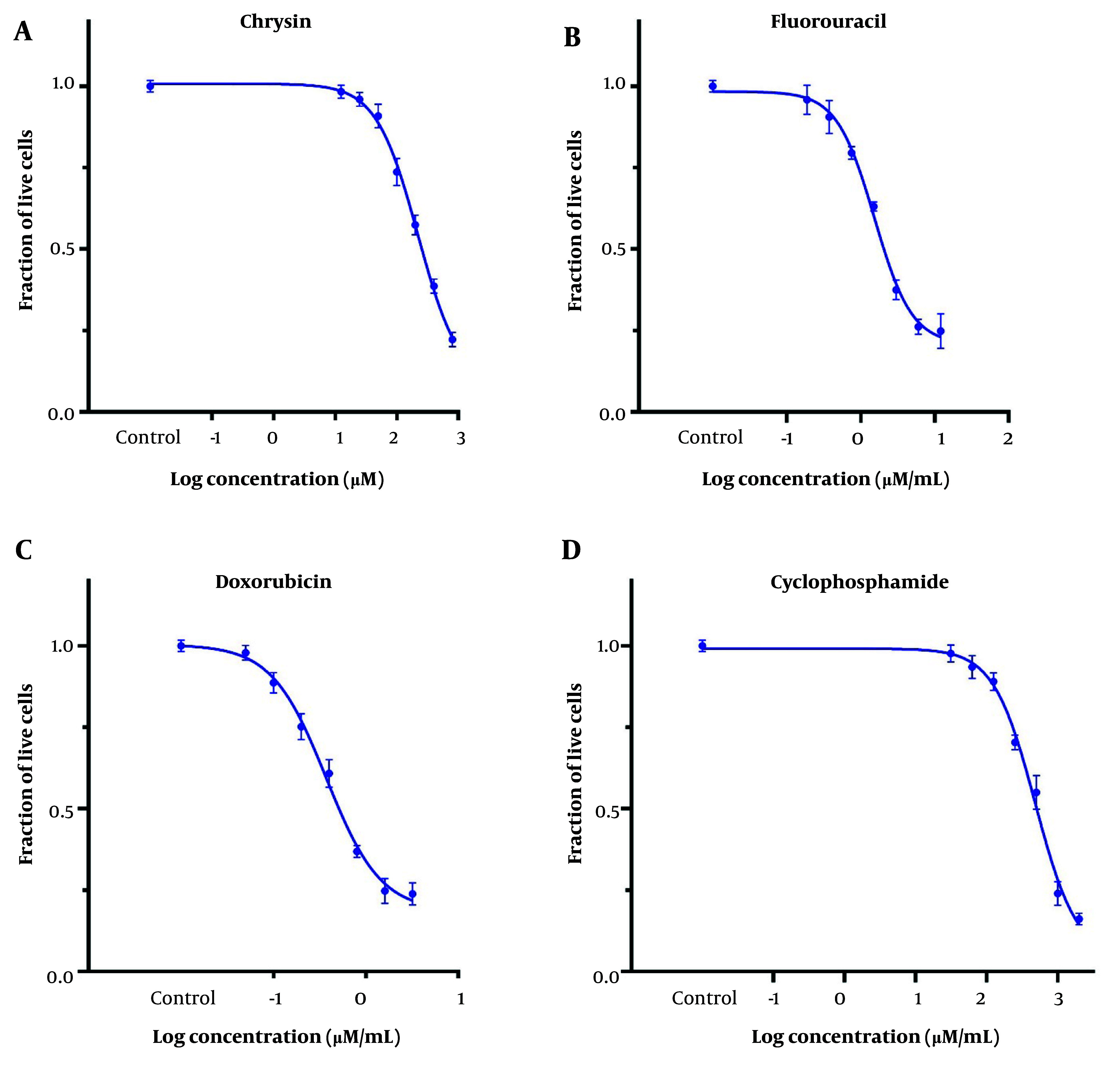
The dose-response curve on MDA-MB 231 cell viability for 48 h; A, chrysin (CH); B, 5-fluorouracil (5-FU); C, doxorubicin (DOX); and D, cyclophosphamide (CP). Each value represents the mean ± SD (n = 3).

Cytotoxicity was investigated using tetrazolium reduction assays with MTT. The IC_50_ values for CH, 5-FU, DOX, and CP were 221 ± 15 μM (56.2 ± 3.8 μg/mL), 11.8 ± 6.9 μM (1.53 ± 0.91 μg/mL), 0.68 ± 0.07 μM (0.37 ± 0.04 μg/mL), and 402.5 ± 17 μM (484 ± 20 μg/mL) on the MDA-MB-231 cell line, respectively (Figure 1). The IC_25_ of the desired drugs was used in subsequent studies (Table 1). 

**Table 1.** The IC_25_ and IC_50_ of the Desired Drugs Were on the MDA-MB-231 Cell Line for 48 h (n = 3)

Drugs	IC_25_		IC_50_	
	μM	μg/mL	μM	μg/mL
**CH**	94 ± 9	23.9 ± 2.3	221 ± 15	56.2 ± 3.8
**5-FU**	6.2 ± 0.4	0.80 ± 0.05	11.8 ± 6.9	1.53 ± 0.91
**DOX**	0.33 ± 0.05	0.18 ± 0.03	0.68 ± 0.07	0.37 ± 0.04
**CP**	192 ± 13	231 ± 15	402.5 ± 17	484 ± 20

Abbreviations: CH, chrysin; 5-FU, 5-fluorouracil; DOX, doxorubicin; CP, cyclophosphamide.

### 4.2. Combination Test by the Checkerboard Microplate Method

The fractional inhibitory indices of the combined application of CH and 5-FU, DOX, and CP were 0.86 ± 0.06, 0.98 ± 0.04, and 1.1 ± 0.07, respectively. Chrysin synergistically enhanced the effects of 5-FU and DOX (FIX < 1) but displayed a non-additive effect with CP (FIX > 1) on the MDA-MB-231 cell line (Table 2). 

**Table 2.** Effects of Chrysin and Anticancer Drugs (5-fluorouracil, doxorubicin, and cyclophosphamide) Interactions on the MDA-MB-231 Cell Line (n = 3)

Drugs	FIX	Comments
**5-FU + CH **	0.86 ± 0.06	Synergistic
**DOX + CH **	0.98 ± 0.04	Synergistic
**CP + CH **	1.1 ± 0.07	Non-additive

Abbreviations: CH, chrysin; 5-FU, 5-fluorouracil; DOX, doxorubicin; CP, cyclophosphamide.

### 4.3. Hemolytic Assay

**Figure 2.**
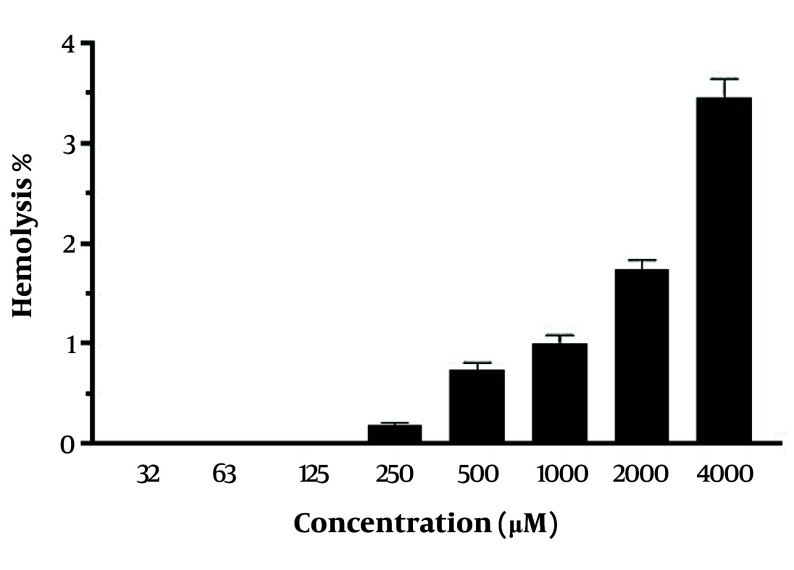
Hemolysis effects of chrysin (CH). Each value represents the mean ± SD (n = 3).

The hemocompatibility of CH was assessed using fresh red blood cells (RBCs) in the CH concentration range of 32 - 4000 μM. No significant hemolytic toxicity was observed at both low and high CH concentrations. At a concentration of 4000 μM, only 3.45 ± 0.19% hemolysis was observed with CH (Figure 2). 

### 4.4. Assessment of Apoptosis by Flow Cytometry

**Figure 3.**
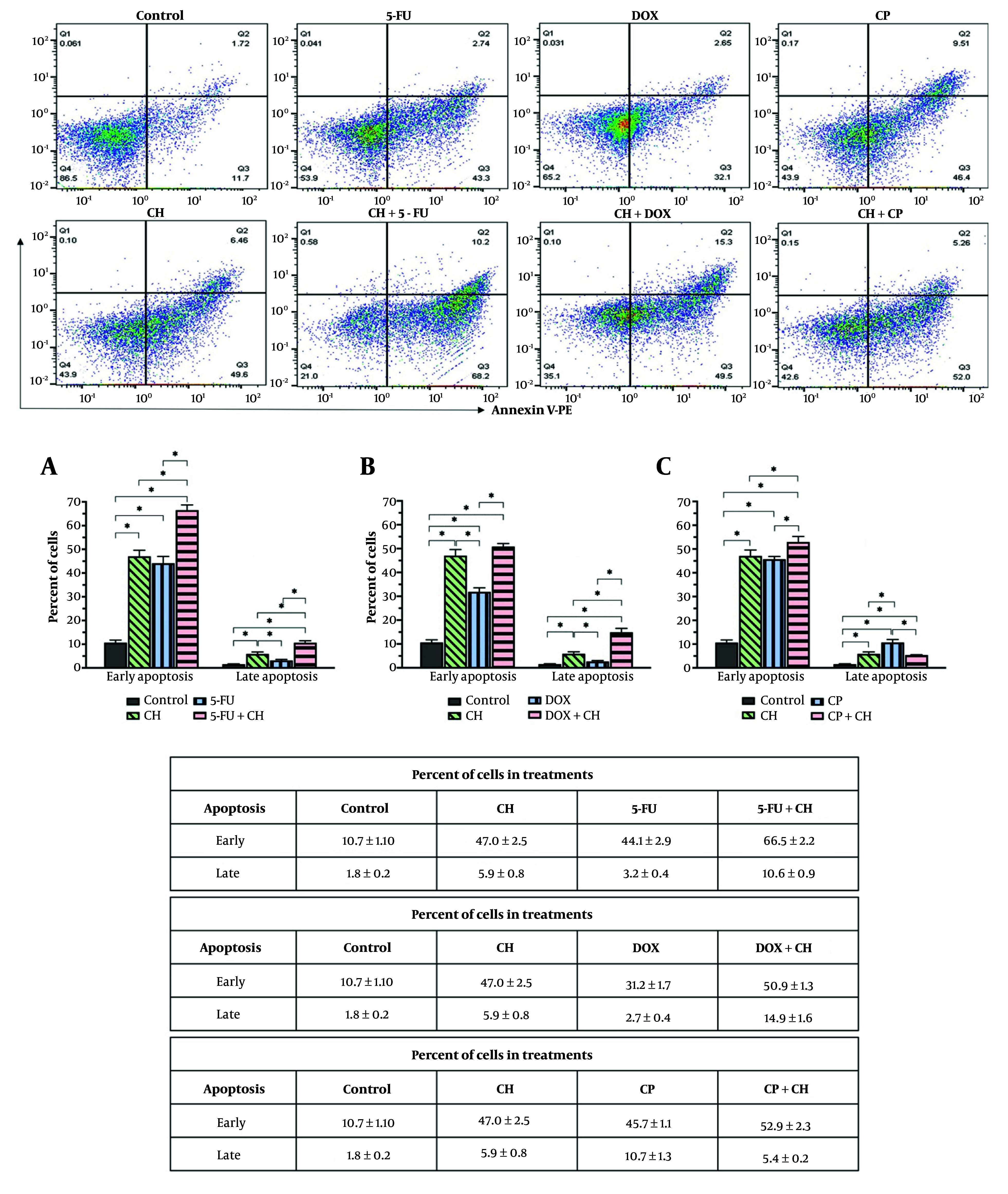
Induction of apoptosis by chrysin (CH) and anticancer drugs [5-fluorouracil (5-FU), doxorubicin (DOX), and cyclophosphamide (CP)]. The combined effect of CH with A, 5-FU; B, DOX; and C, CP in early and late apoptosis on the MDA-MB 231 cell line. Each value represents the mean ± SD (n = 3). Data were analyzed using one-way analysis of variance (ANOVA) with Tukey's test for multiple group comparisons. * P < 0.05.

Chrysin was combined with each chemotherapeutic drug at the IC_25_ dose to investigate drug interactions on the MDA-MB-231 cell line. As shown in Figure 3, the combination treatment of CH and each chemotherapeutic drug was more effective in inducing early and late apoptosis on the MDA-MB-231 cell line compared with the control (P < 0.05). According to ANOVA analysis, the combination treatment of CH with 5-FU significantly induced early and late apoptosis compared with CH and 5-FU treatments alone (P < 0.05). The combination of CH with DOX significantly increased late apoptosis compared to DOX and CH alone (P < 0.05). The combination of CH with CP significantly increased early apoptosis compared to either drug used alone (P < 0.05).

### 4.5. Evaluation of Cell Cycle Status by Flow Cytometry

**Figure 4.**
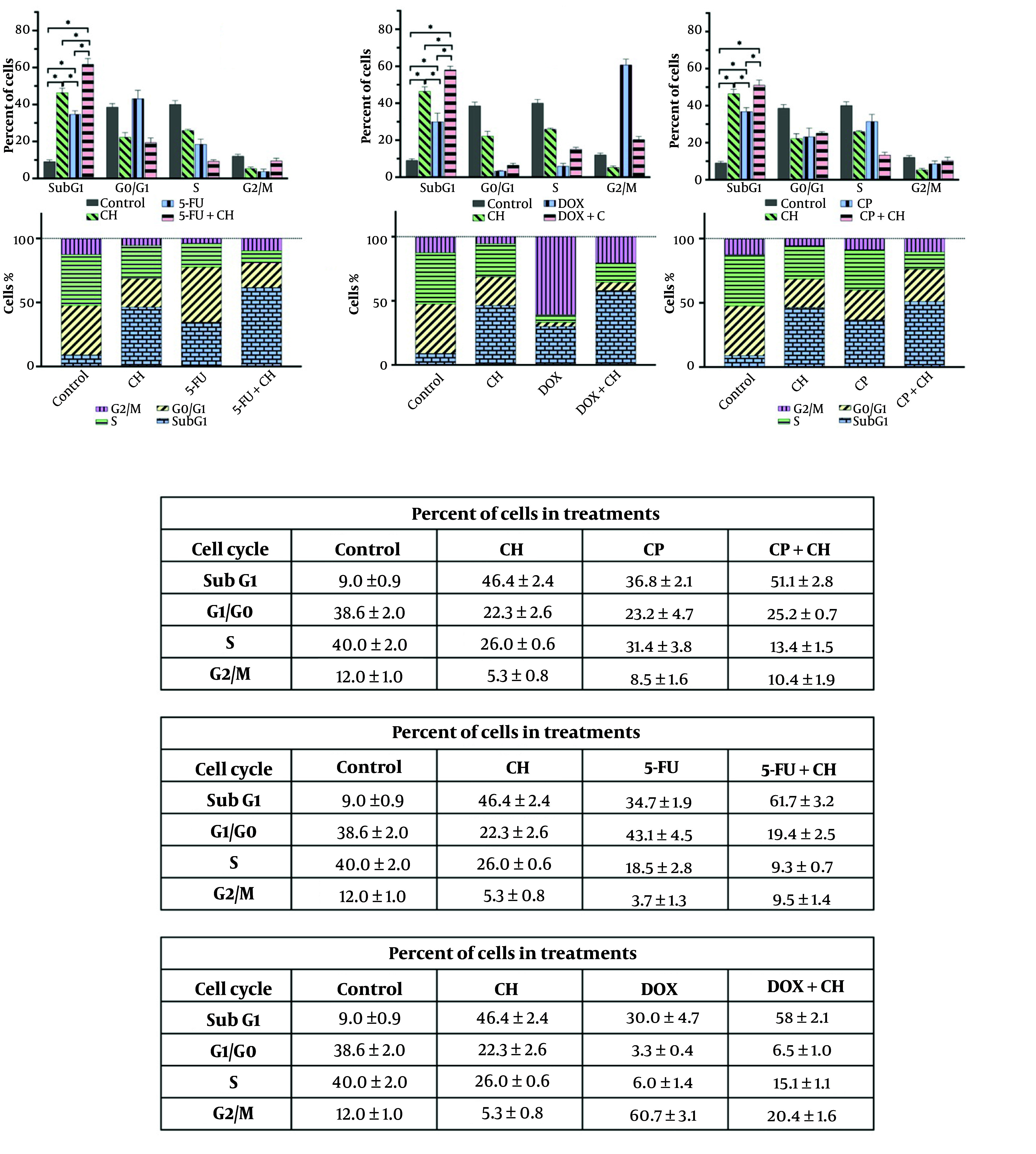
Cell cycle analysis of chrysin (CH) with A, 5-fluorouracil (5-FU); B, doxorubicin (DOX); and C, cyclophosphamide (CP) on the MDA-MB 231 cell line. Each value represents the mean ± SD (n = 3). Data were analyzed using two-way analysis of variance (ANOVA) with Tukey's test for multiple group comparisons. * P < 0.05.

Treatment with CH, 5-FU, DOX, and CP, both individually and in combinations, significantly increased the sub-G1 phase compared to the control (P < 0.05). The combinations of CH with 5-FU or DOX further elevated the sub-G1 phase compared to the individual drugs (P < 0.05), and CH with CP showed a significant increase over CP alone (P < 0.05) (Figure 4). 

### 4.6. Cell Migration

**Figure 5.**
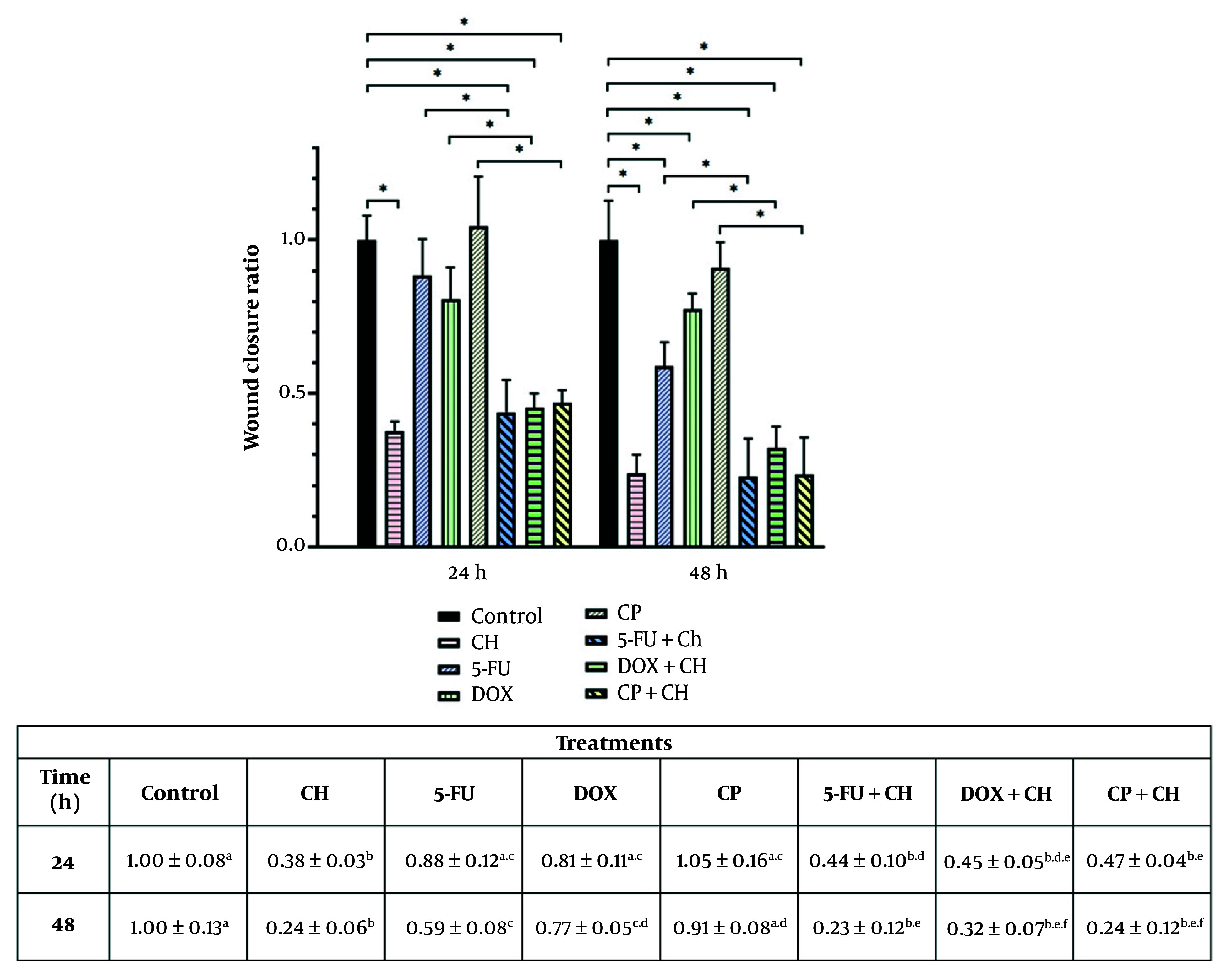
Migration assay of chrysin (CH) and chemotherapeutic drugs [5-fluorouracil (5-FU), doxorubicin (DOX), and cyclophosphamide (CP)] in combination or alone on the second day (24 h) and third day (48 h) on the MDA-MB-231 cell line. Each value represents the mean ± SD (n = 4). Data were analyzed using two-way analysis of variance (ANOVA) with Tukey's test for multiple group comparisons. Means in the same row with different superscripts are significantly different (P < 0.05). * P < 0.05. Means in the same row with different superscripts are significantly different (P < 0.05).

A migration assay was performed to assess the effect of CH and chemotherapeutic drugs, both in combination and alone, on cell migration. As presented in Figure 5, cell migration significantly decreased upon treatment of MDA-MB-231 cells with CH at both 24 and 48 hours, and with 5-FU and DOX at 48 hours, compared with the control (P < 0.05). The combination of CH with 5-FU, DOX, and CP significantly decreased cell migration compared with 5-FU, DOX, and CP alone on MDA-MB-231 cells (P < 0.05).

## 5. Discussion

The limiting effectiveness of chemotherapeutic drugs in cancer treatment due to drug resistance and systemic toxicity remains a significant challenge for successful cancer treatment. However, due to the lack of estrogen and progesterone receptors and HER2 expression, TNBC exhibits no responsiveness to the major available treatments, leaving conventional chemotherapy as the only standard treatment option (17). A promising strategy to overcome chemotherapy resistance involves combining chemotherapeutic drugs with chemical compounds like flavonoids, which have additive effects. Specifically, CH has demonstrated various anti-cancer properties (10, 16-18).

We evaluated the effects of co-treating MDA-MB-231 cells with CH and the chemotherapeutic agents 5-FU, DOX, and CP. Our findings indicated that 5-FU and DOX synergized with CH, whereas CH and CP exhibited an antagonistic effect. The results also showed a low hemolytic percentage (up to 3.4%) at concentrations ranging from 32 to 4000 μM. In contrast, Halevas et al. reported that CH induced hemolytic activity higher than 5% at concentrations between 400 and 2000 μM (18). According to the American Society for Testing and Materials (ASTM) E2524-08 (2013) standard, a hemolysis percentage greater than 5% indicates damage to RBCs (19). The variation in hemolytic activity with CH treatment might be attributed to different blood groups of the ABO system, which have specific antigens resulting in distinct biochemical properties (20).

Our findings confirm that CH, 5-FU, DOX, and CP induce significant apoptosis (21). Moreover, combining CH with 5-FU or DOX is more effective at inducing apoptosis in MDA-MB-231 cells than using the drugs alone. The intricate apoptotic dysfunction in TNBC enables cells to evade apoptosis, leading to resistance against critical cytotoxic agents such as DOX and CP (6, 16). Yong et al. indicated that CH blocks TNBC growth and proliferation by inhibiting the PI3K/Akt/mTOR signaling pathway, crucial for cancer cell survival and the regulation of apoptosis-related gene expression (22). Chrysin induces apoptosis in HepG2 cells via the p53/Bcl-2/caspase-9 pathway (23). Zheng et al. demonstrated that 5-FU activates caspases and p53 while inhibiting CDK-2 in BT-549 and MDA-MB-231 cells (24).

5-Fluorouracil induces apoptosis in breast cancer cell lines by phosphorylating anti-apoptotic genes and upregulating pro-apoptotic markers (25). Doxorubicin similarly causes cell death through DNA intercalation, topoisomerase trapping, reactive oxygen species (ROS) generation, and upregulation of immune-related genes such as PD-1/PD-L1 (26). The study links cell cycle distribution to apoptosis, showing that CH, alone or in combination with 5-FU, DOX, or CP, increases apoptotic cells in the sub-G1 region. Chrysin induces G1 cell cycle arrest by raising p21 (Waf1/Cip1) levels and reducing CDK2/cyclin E and CDK4/cyclin D activity (27). The combination of CH and 5-FU enhances anti-cancer effects by causing G2/M phase arrest in 5-FU-resistant AGS cells. 5-fluorouracil exerts cytotoxicity by misincorporating into DNA and RNA, leading to DNA damage and inhibiting dTMP production (28). Co-administration of CP and vincristine effectively kills pancreatic cancer cells in the G1 phase (29). Cyclophosphamide increases p53, p16, and γ-H2AX levels in 4T1 cells and induces ROS production (30). In MDA-MB-231 cells, co-treatment with CH and DOX shifted cell cycle progression from G2/M arrest to an increased sub-G1 population. Our findings align with the reports by Sabzichi et al., which showed that CH increases the efficacy of DOX by altering cell cycle distribution in MCF-7 cells via inhibition of the Nrf2 pathway (23). We found that CH inhibits MDA-MB-231 cell migration and enhances the efficacy of 5-FU, DOX, and CP. Yang et al. reported that CH pretreatment in TNBC cells promotes anti-metastatic activity by inhibiting MMP-10 and Akt signaling pathways (31). To our knowledge, the present study is the first report on the anti-tumor effect of combining CH with routine chemotherapy drugs, including 5-FU, DOX, and CP, on MDA-MB-231 cells. Nevertheless, the fundamental mechanism has yet to be ascertained, and thus, additional investigations are requisite. One limitation of this investigation is that it was restricted solely to exploring the cellular level. Moreover, the lack of normal cell use could also be another limitation of the present study.

### 5.1. Conclusions

The results revealed that combining CH with 5-FU and DOX exerted a more potent therapeutic impact on TNBC cells than individual treatments. This enhanced effect was achieved through the induction of apoptosis, cell cycle arrest in the sub-G1 phase, and inhibition of cell migration. The synergistic impact of CH with these chemotherapy drugs effectively inhibits the proliferation of cancer cells, ultimately improving their therapeutic efficacy.

## Data Availability

The dataset presented in the study is available on request from the corresponding author during submission or after publication.
